# Multistable Perception in Older Adults: Constructing a Whole from Fragments

**DOI:** 10.3390/brainsci6010010

**Published:** 2016-03-22

**Authors:** Khushi Patel, Maureen Reed

**Affiliations:** Department of Psychology, Ryerson University, Toronto, ON M5B 2K3, Canada

**Keywords:** multistable perception, visual perception, optical illusions, cognition, aging

## Abstract

Visual perception is constructive in nature; that is, a coherent whole is generated from ambiguous fragments that are encountered in dynamic visual scenes. Creating this coherent whole from fragmented sensory inputs requires one to detect, identify, distinguish and organize sensory input. The organization of fragments into a coherent whole is facilitated by the continuous interactions between lower level sensory inputs and higher order processes. However, age-related declines are found in both neural structures and cognitive processes (e.g., attention and inhibition). The impact of these declines on the constructive nature of visual processing was the focus of this study. Here we asked younger adults, young-old (65–79 years), and old-old adults (80+ years) to view a multistable figure (*i.e.*, Necker cube) under four conditions (free, priming, volition, and adaptation) and report, via a button press, when percepts spontaneously changed. The oldest-olds, unlike young-olds and younger adults, were influenced by priming, had less visual stability during volition and showed less ability to adapt to multistable stimuli. These results suggest that the ability to construct a coherent whole from fragments declines with age. More specifically, vision is constructed differently in the old-olds, which might influence environmental interpretations and navigational abilities in this age group.

## 1. Introduction

Visual perception is constructive in nature; that is a coherent whole is generated from ambiguous fragments that are encountered in dynamic visual scenes. Creating this coherent whole from fragmented sensory inputs requires one to detect, identify, distinguish and organize sensory input [1]. The organization of fragments into a coherent whole is facilitated by the continuous interactions between lower level sensory inputs and higher order processes. However, age-related changes are observed in both, early sensory brain regions (*i.e.*, area V1) as well as non-sensory higher order associative cortices (*i.e.*, prefrontal) [2,3,4]. These age-related changes can alter the communication between lower level and higher order brain regions, and may disrupt the constructive process of visual perception, which might in turn interfere with older adult’s ability to interpret the ambiguities present in their environment [1]. Understanding this constructive process in older adults is the purpose of the current paper.

One way to investigate the communication between lower level and higher order regions is through the use of multistable perception. Multistable perception occurs when sensory information is ambiguous, and two or more distinct interpretations of a single sensory input are available to the viewer in quick succession. Multistable stimuli (e.g., Necker Cube), are missing some cues or fragments necessary for constructing a coherent whole and as a result neural activity in the sensory regions (*i.e.*, primary visual cortex) fluctuates causing perceptual instability, which in turn encourages higher-order regions (*i.e.*, frontal and parietal cortices) to re-evaluate and initiate a perceptual reorganization (*i.e.*, perceptual switch or reversal) [1]. These stimuli offer the opportunity to investigate the interactive nature of higher and lower perceptual and cognitive mechanisms during visual construction, within a controlled laboratory setting.

Multistable perception has been explained by both bottom up and top-down theoretical approaches. However, available ERP and neuroimaging evidence supports both approaches and as such most researchers suggest a combined or hybrid model to explain multistable perceptual outcomes [1,5,6,7,8,9]. ERP data shows two components related to reversals; a reversal negative (RN) component and a late positive component (LPC; [10,11,12]). The RN component appears to be associated with early bottom up processing [10,12], and Pitts *et al.* [10] found that this component was associated with activity in the inferior occipital-temporal cortex, while the LPC component is associated with activity in the inferior temporal and superior parietal regions. Russo and De Pascalis [13] suggest the late component represents a stage where the ambiguous figure is internalized; perhaps indicating a top-down process. Regardless, the hybrid model indicates that perceptual switches during multistable perception rely on the interactions between low-level sensory signals and high-level cognitive factors, which take place in the extrastriate visual cortex [10]. Studies utilizing reversible figures (*i.e.*, Rubin’s face-vase illusion) have confirmed the direct involvement of extrastriate visual areas in resolving visual ambiguities and conflicts [1]. Further, percept specific electrophysiological activity in area V5, medial superior temporal and parietal cortex was observed in monkeys during bistable motion perception [14]. Similarly, fMRI signals recorded in humans demonstrated more activity in area V5 and the medial temporal areas during ambiguous figure viewing, especially while perceiving apparent motion in ambiguous stimuli (i.e. switching between two possible percepts) [15]. According to Muckli *et al.* [15], such activation suggests that the medial temporal regions play a mediatory role in the constructive process of human perception.

Age-related decline in both, early sensory brain structures as well as non-sensory higher order associative cortices can interfere with older adults’ ability to interpret the ambiguities present in their everyday environment [1,4]. Structural and physiological changes in the human visual pathways are part of normal aging. These age-related changes at the retinal level can impair older adults’ contrast and spatial sensitivities, as well as their ability to detect the direction of motion even under well-lit conditions [4]. Research also suggests age-related structural changes in the visual pathways beyond the retina. Single unit recordings in area V1 of monkeys has revealed that relative to young monkeys, cortical neurons in old monkeys are less sensitive to contrast, and have a lower spatial resolution and higher temporal frequency thresholds [16,17]. Furthermore, compared to area V1, a pronounced increase in visually driven activity and a decrease in signal-to-noise ratio was observed in area V2 of older monkeys [18], and neurons in area V2 have shown to play an important role in organizing and responding to complex stimuli, such as the orientation of illusory stimuli [19,20]. In humans, age-related neuronal death may result in a noisy visual system, which in turn affects their ability to perform everyday task that rely heavily on vision (*i.e.*, mobility) [4].

The ability to process (*i.e.*, recognize, distinguish, identify) sensory inputs also involves higher-order regions (*i.e.*, frontal and parietal cortices). Age-related changes in the prefrontal and parietal cortices play a crucial role in cognitive aging (*i.e.*, deficits in attention, inhibition, working memory), resulting in behavioral changes (*i.e.*, slow visual processing speed, deficits in inhibition and attention switching) [4,21]. According to Hasher *et al.* [2], inhibition narrows the focus of working memory, which results in faster processing of information. The frontal lobe structures play a critical role in inhibition, importantly; prefrontal cortex structures are associated with inhibition and in structural brain studies these structures show the largest deficits due to age [2]. In addition, there are age-related changes in the sub cortical structures and neurotransmitter systems that communicate with the prefrontal cortex during the process of inhibition. Neurotransmitter dopamine and norepinephrine maintain task relevant information and prevent irrelevant information from becoming active, however, functioning of these neurotransmitters show variations with age, in that concentration of these neurotransmitters decrease in certain brain regions as a result of neuronal aging [2]. Overall, the ability to suppress irrelevant sensory information is vital for efficient environmental performance. Particularly during multistable perception, perceptual dominance fails to settle in a stable state and it fluctuates unpredictably with prolonged viewing, resulting in perceptual reversals experienced while viewing illusory stimuli [22]. Inhibition and neural adaptation play a major role in this process of competing neural representations, which allows the dominant percept to gain the upper hand. Additionally, spontaneous neural discharge (neural noise) increases with age, which may result in decrements in visual perception, short-term memory, motor skills and other cognitive functions [3].

There is limited research on multistable perception in older adults. Some early researchers suggested that there was little effect of aging on multistable perception using the Necker Cube [23]. Yet these and other researchers have since suggested that the null effect was due to sampling bias [23,24]. Most available research suggests that, in general, with multistable illusions, younger adults see more perceptual switches than do older adults [21,24,25] and that older adults spend more time in the dominant perspective (*i.e.*, length of time between reversals) than do younger adults [26]. Further, Heath and Orbach [27] found that many older adults, unlike their younger counterparts, are incapable of switching perspectives of the Necker cube. Older adults may have an inability to voluntarily hold the dominant percept for a prolonged period of time [21]. Aydin *et al.* [21] suggest it is the decline in attention mechanisms that impairs older adult’s ability to switch and hold percepts of multistable illusions. Yet, given that younger adults’ voluntary attentional control over multistable perception is limited, Sterzer *et al.* [1] suggests that voluntary attentional control might actually serve as feedback that could initiate reevaluation and reorganization of the perceptual reconstructive processes.

Multistable perception exemplifies the interpretive nature of the visual system and studying multistable stimuli can aid in understanding the constructive nature of the visual system. More specifically, conducting research with older adults using multistable illusions like the Necker cube will help to address questions related to the communication between sensory and cognitive processes. The current study builds upon the research presented above. More specifically, here we investigated the effects of age-related deficits at the sensory and cognitive level on visual construction using multistable stimuli. We manipulated our experimental paradigm to examine the impact of age-related changes in sensory, attention and inhibition abilities on perceptual outcomes during multistable viewing. In addition, we uniquely examine outcomes of young-olds (65–79 years) as compared to old-olds (80+ years) in order to determine the effect of continued age-related losses on visual construction.

## 2. Materials and Methods

### 2.1. Participants

This study included 3 age groups; 20 younger adults (18–30 years), 20 young-olds (60–79 years) and 20 old-olds (80+ years). The younger adult group was recruited from a university Undergraduate research pool while the older adults were recruited from a senior participant pool, which is a secured internal database of research older adult volunteers housed at the institution of the authors. All participants completed 2 visual screening tests (*i.e.*, Regan vision letters charts 96% and 11%, and Stereo Acuity test) prior to the experimental procedure. All older adults were healthy community dwelling, high functioning individuals who lived independently. While they were not screened for dementia, all self-reported good physical and cognitive health and arrived to the laboratory independently. The young-olds and old-olds groups received a compensation of $15 and the younger adults received 1 credit towards their Undergraduate introductory psychology course.

Participants included younger adults (M = 20.12, SE = 0.49), young-old adults (M = 71.4 years, SE = 0.53) and old-old adults (M = 84.08, SE = 0.66). Mean acuities were within the normal range for all groups (20/30 or better). Based on confidence (95%), older adults in both groups showed significantly poorer contrast and depth ability, relative to younger adults. Two thirds of the younger adults and young-old adults were female, while about half of the old-old adults were female. About one-third of younger adults wore corrective lenses (all <1.5 diopters or less), while almost all of the older adults in this study wore corrective lenses and these individuals wore these lenses during testing. Only two older adults (one young-old and one old-old) scored acuities outside the normal range with their spectacle correction (one scored 20/50 and the other 20/100 respectively). However, performance of these individuals on our experimental tasks was within the central 95% of their age group. Table 1 shows the mean visual demographics for each age group.

### 2.2. Apparatus

#### 2.2.1. Regan Vision Letter Charts

The letter chart presented rows of black letters on a white background. The rows of letters decreased in size as one progressed down the chart. Acuity was measured by the smallest row of letters that can be reliably read from 10 feet distance. All participants viewed 2 charts (96% and 11% contrast).

#### 2.2.2. Stereo Acuity Test

Depth perception was measured using the Stereo Circle Test (Stereo Optical Company, Inc., Chicago, IL, USA). The participants were asked to wear stereo filter glasses while viewing 9 successive stereo dot images and identify which dot image was raised above the others in a group of four dots. The disparity progressively reduces on successive images. The smallest disparity that evoked depth was taken as a measure of stereo acuity.

#### 2.2.3. Necker Cube

**Figure 1:**
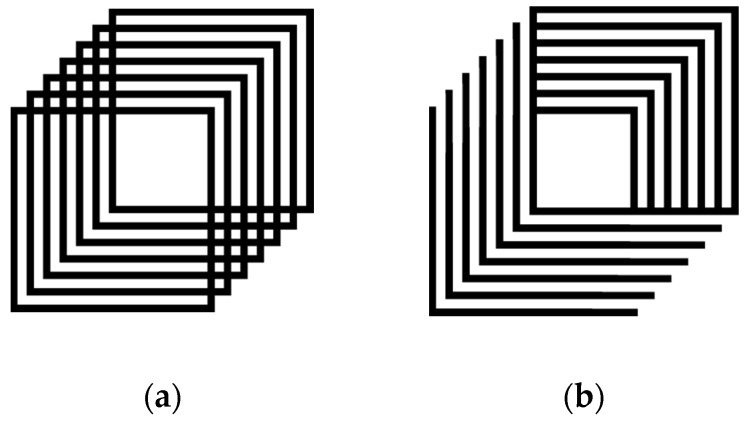
Necker cube stimuli. (**a**) Necker cube, two or more distinct percepts can be viewed; And (**b**) unambiguous prime and adaptation cube.

The Necker cube is an example of a multistable perception, as two or more distinct percepts can be viewed spontaneously for this single stimulus. The orientation of the Necker cube was manipulated and designed in a way that it was strictly either ambiguous or unambiguous. The Necker cube (see Figure 1a) allowed the participants to see both possible orientations (down to the left or up to the right) and thus was labelled ambiguous. The cube used for priming and adaptation is shown in Figure 1b. While this cube appears similar to the Necker cube, its closure allows for only one perspective; up to the right and is thus referred to as the unambiguous cube.

### 2.3. Design and Procedure

Once this study received ethical review and approval, participants were recruited. After signing a consent form, each participant completed two visual screening tests (letter acuity and depth perception) prior to starting the experiment. Participants were then familiarized with the two possible percepts of the Necker cube (*i.e.*, down to the left and up to the right, see Figure 1a) and once they understood, they were asked to complete four computer-based tasks.

#### 2.3.1. Experiment 1: Baseline Measurements

Participants were asked to view a Necker cube (see Figure 1a) for 3 min in total over six exposures (30 s per exposure) and indicate by pressing an assigned key on the keyboard when the cube changed perspective. The data gathered, in this and all following experiments included the number of reversals reported by key press and the number of milliseconds spent in each orientation. These data created a baseline of performance for each of the three groups.

#### 2.3.2. Experiment 2: Priming

Participants were asked to view the unambiguous cube (Prime, see Figure 1b) for 5 s followed by a 30 s viewing of the Necker cube and report when the cube changed perspective via a key press. This task was repeated 6 times.

#### 2.3.3. Experiment 3: Volition

Participants were instructed to view a Necker cube and hold its perspective in up to the right orientation (30 s for each exposure). During this task, participants were asked to press the assigned keys and indicate if any reversals in their percept occurred while they tried to hold the view of the Necker cube in one orientation. This task was repeated 6 times.

#### 2.3.4. Experiment 4: Adaptation

Participants were instructed to fixate on a cross in the center of an unambiguous cube (see Figure 1b) for 1 min followed by a 30 s viewing of a Necker cube. While viewing the Necker cube the participants were asked to indicate if reversals occurred via key press. This task was repeated 6 times.

## 3. Results

**Figure 2:**
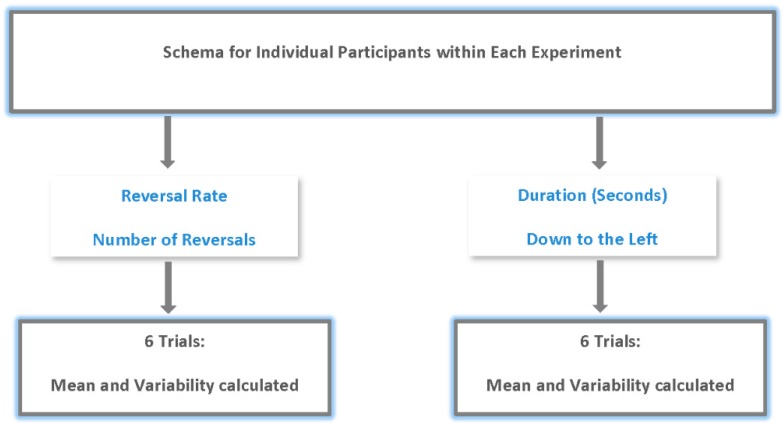
Schema of Individual participant trials

The purpose of this study was to examine changes in the constructive nature of vision with age. To this end, the processing of multistable figures (Necker cube) across age groups was examined. Two dependent measures were used (see Figure 2). The first was the number of Necker cube reversals reported (by button press) by individuals. For each participant the number of reversals reported (via button presses) was averaged over their six repetitions within each experiment. This measure allowed us to examine the changes in the way age affects perceptual conflict. Participants provided multiple responses, as noted above, in each experimental condition. Thus, the average reversals and the precision of reversal response over six trials (variability) were analyzed for each experiment. The second measure was the duration of the trial spent in a single Necker cube orientation. Here we measured the amount of time in seconds in the “down to the left” orientation over six 30 s exposures. The amount of time was then averaged for each individual over their six exposures for each experiment. This measure was important because it is possible that even with large numbers of reversals an individual may spend more time in one orientation than the other and this may have implications for neural and higher order processing. Since participants provided the “down to left” orientation response multiple times in each condition, we also measured the precision of response (variability) and this too was analyzed.

Analysis of Variance procedures were used to determine group differences (mean response and precision) in number of reversal reported and time spent in a single orientation, followed by post-hoc comparisons. In addition, correlation was used to examine the relationship between dependent measures and age and vision status.

### 3.1. Does Multistable Perception Differ between Older and Younger Adults (Baseline Measures)?

Participants were asked to view the Necker stimulus and indicate with a button press when it changed orientations. Overall, a significant effect of age was found on the number of Necker cube reversals reported (F_2, 72_ = 4.125, *p* = 0.02, Eta = 0.103). Post hoc Tukey Tests (*p* < 0.05) showed that the younger adults had significantly more reversals (M = 8.4, SE = 3.81) over thirty second periods than did the old-old adults (M = 5.2, SE = 4.07). Young-old adults showed no significant difference in the number of reversals (M = 6.6, SE = 3.99) from younger adults. There were no significant differences in the amount of time spent in one orientation of the Necker cube between the three age groups (F_2, 72_ = 0.84, *p* = 0.44).

All participants provided multiple responses for each dependent measure. Analysis of the precision (variability) of their responses over presentations indicated that there were no significant age differences in the perceptual stability of reversal rate or time spent in one orientation (all *p* > 0.05).

### 3.2. Are There Differences between Age Groups Due to the Influence of Priming on Multistable Perception?

Participants were briefly shown (5 s) an unambiguous priming cube (see, Figure 1b) in the “up to the right” orientation prior to viewing the multistable stimulus (Necker cube, see Figure 1a) for 30 s. Post-priming responses were compared to initial (baseline) responses. Both reversal rate and the time in a single orientation were measured. After exposure to the prime there was a small reduction in the number of reversals reported across all groups (F_1, 72_ = 6.9, *p* = 0.01, Eta = 0.09) and overall both older adult groups showed fewer reversals than did younger adults (F_2, 72_ = 6.72, *p* = 0.002, Eta = 0.157, both Tukey *p* < 0.05). However, there was no significant interaction, suggesting that the prime did not uniquely affect the reversal rates differently between older and younger adults (F_2, 72_ = 0.76, *p* = 0.47). Nor did the prime affect the stability of responses over multiple presentations of the Necker cube (all F, *p* > 0.05).

**Figure 3:**
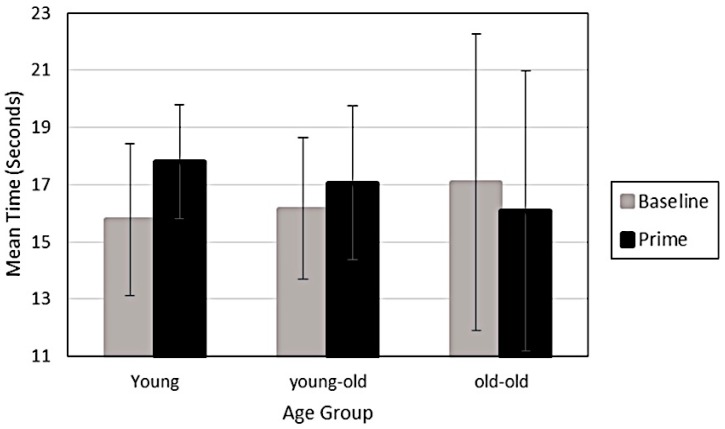
Mean time and standard error for all 3 age groups in the “down to the left” orientation at baseline and then after Priming, where the prime cube in the “up to the right” orientation was viewed for 5 s prior to viewing the Necker cube.

However, a significant interaction indicated that the old-old adults decreased their time in the orientation opposite of the prime following exposure, while young-old and younger adults slightly increased their time in the orientation opposite of the prime following exposure (F_2, 72_ = 3.6, *p* = 0.03, Eta = 0.09). In other words, as shown in Figure 3, only for the old-old adults did the prime influence perception towards the orientation of the prime. The prime influenced the young-olds and younger adults towards the orientation opposite of the prime. This suggests that even short exposures of five seconds influenced younger and young-old adults to adapt to the prime. Further, during the priming experiment old-old adults were significantly less stable (precision) in their responses over trials than younger adults (F_2, 72_ = 3.82, *p* = 0.03, Eta = 0.10, Tukey, *p* < 0.05).

### 3.3. Are their Differences between Age Groups in the Ability to Hold Perception in One Orientation?

Participants were asked to hold the perception of the Necker cube in the “up to the right” orientation (called volition). This instruction led to a decrease in the number of perceptual reversals experienced for all age groups (F_1, 72_ = 7.61, *p* = 0.01, Eta = 0.09) with the old-olds continuing to experience fewer reversals than younger adults (F_2, 72_ = 4.09, *p* = 0.02, Eta = 0.10, Tukey, *p* < 0.05). However, no significant interaction was revealed suggesting that volition did not uniquely affect the reversal rates of any age group (F_2, 72_ = 1.31, *p* = 0.28). Further, no changes in the stability (precision) of reversal rates over trials were found for any age group relative to baseline measures (all F > 0.05).

**Figure 4:**
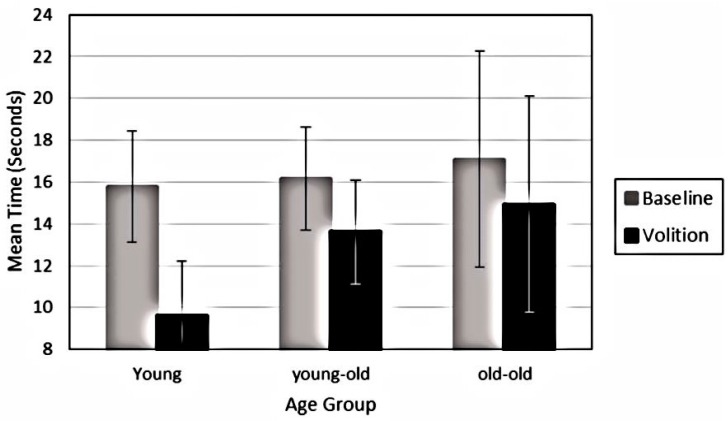
Mean time and standard error in the “down to the left” orientation and following instructions to hold the image in the “up to the right” orientation for younger adults, young-old and old-old adults.

The requirement to “see” the cube in the up to the right orientation, however, had a significant impact on time spent in the down to the left orientation. Significant decreases were found in experiencing the down to the left orientation for all age groups (F_1, 72_ = 47.93, *p* < 0.001, Eta = 0.40). Yet, as illustrated in Figure 4, a significant interaction indicated that younger adults showed significant reductions in seeing the cube in the down to the left orientation, while only mild reductions were found for both older adult groups (F_2, 72_ = 6.05, *p* = 0.004, Eta = 0.144). Volition also led to less stability of perception over trials for all groups (F_1, 72_ = 5.41, *p* = 0.02, Eta = 0.070), and in particular the old-old adults were less stable in their perception than younger adults (F_2, 72_ = 4.99, *p* = 0.01, Eta = 0.122, Tukey *p* < 0.05).

### 3.4. Are There Differences between Age Groups on the Effect of Adaptation on Perceptual Orientation?

Participants were briefly shown (1 min) the unambiguous cube (oriented up to the right; Figure 1b) prior to the multistable stimulus (Necker cube for 30 s, see Figure 1a). Post adaptation responses were compared to initial (baseline) responses. Overall, fewer reversals were found for all groups after adaptation, relative to baseline (F_1, 72_ = 11.65, *p* = 0.001, Eta = 0.14) and old-old adults continued to show fewer reversals than did younger adults (F_2, 72_ = 4.74, *p* = 0.012, Eta = 0.12, Tukey, *p* < 0.05). However, no significant interaction was revealed suggesting that adaptation did not uniquely affect the reversal rates of any age group (F_2, 72_ = 0.14, *p* = 0.87). Further, no changes in the stability of reversal rates over trials were found for any age group or relative to baseline measures (all F > 0.05).

In contrast, time spent in the ‘down to the left” orientation significantly increased after adaptation (F_1, 72_ = 60.2, *p* < 0.001, Eta = 0.46) and a significant interaction suggested that there was a larger effect of adaptation on younger and young-old adults than on old-old adults (F_2, 72_ = 3.326, *p* = 0.042, Eta = 0.09). Overall, participants in all age groups showed less stability in their responses after adaptation (F_1, 72_ = 5.06, *p* = 0.03, Eta = 0.07), and the old-olds were significantly less stable in their perception than were younger adults (F_2, 72_ = 6.151, *p* = 0.003, Eta = 0.15, Tukey, *p* < 0.05).

### 3.5. Correlations

While no significant correlations were found between vision (acuity, contrast or depth abilities) and baseline reversal rate or baseline time spent in one orientation, a few small but significant correlations were found in the priming, volition and adaptation phases of this experiment. For acuity, the young-olds with poorest acuity had stronger adaptation effects in the priming condition, that is more time was spent in the orientation opposite to the prime (*r* = 0.43, *p* = 0.03). For depth, the young-olds with the poorest depth scores spent the largest times viewing the Necker cube in the orientation opposite to the required orientation during volition (*r* = 0.45, *p* = 0.02) and those young-olds with the poorest depth had the highest number of reversals and least stability in the adaptation condition (*r* = 0.56 and 0.43, respectively, *p* < 0.04). The old-olds with the poorest depth showed the least stability in the prime condition (*r* = 0.57, *p* = 0.003).

## 4. Discussion

In this study we examined the effects of age on the constructive nature of vision. Overall we found that the old-olds have more difficulties resolving ambiguities than do other age groups and this may be due to both attentional and neural changes. Unlike younger counterparts, old-olds are influenced in construction by external information sources, and they are less able to consistently inhibit these external sources during construction. Further, unlike the younger adult and young-old age groups, the old-olds have less ability to adapt and are less stable in adaptation across trials. In contrast to these findings, the current study demonstrated many perceptual similarities between younger adults and young-olds (65–79 years of age). The young-olds were similar to younger adults in their baseline patterns of responding, in their effect of the prime acting as an adapting stimulus (though they did show fewer reversals), in their stability of perceptual orientation during volition (though they did show less time in the required orientation) and in the effects of the adapting cube on their percept. Yet, unlike younger adults and old-olds (except for priming), the young-olds’ responses were correlated with their acuity and depth abilities.

Differences found between the old-olds and their young-old counterparts may be in part due to age-related changes in the signal to noise ratios. In the current study, the old-olds were less stable in their percepts during priming, volition, and adaptation conditions suggesting perceptual stability losses that impact on both sensory and cognitive (attention; inhibition) function. Single cell studies in very old macaque monkeys have shown that age-related degradation in the early visual processing regions (area V2) leads to decreased selectivity and signal to noise ratios for direction and orientation tasks [18]. Changes in the signal to noise ratio may account for general losses in perceptual stability. However, the old-olds also were more influenced in their perception by external sources of information (prime), were less able to inhibit (volition) and less able to adapt than their younger counterparts. Bennett *et al.* [28] showed that older adults, relative to their younger counterparts, were less sensitive to motion signals generally and less able to identify the direction of motion. However, age-related losses in direction identification persisted when they accounted for motion sensitivity. Further, these effects of aging were only found to emerge gradually across the lifespan and were pronounced in the old-old participants (70–81 years of age) and were attributed to both neural noise and losses in directional selectivity mechanisms. Relative to the current experiment, our Necker stimulus involves both motion mechanisms during perceptual switches and positional information (orientation of the cube). It is possible then that the differences observed in old-old adults in the present study are due to their inefficiency at positional information (orientation detection), rather than an ability to detect motion during perceptual switches. Increased time at detecting constantly changing positional information may be attributed to the possible degradation in processing positional information early in the visual processing chain, which then impacts these later processes of attention and inhibition.

In support of this suggestion, previous research shows that visual functions in younger adults are related to the size of early visual processing areas (V1, V2 and V3), and there are significant age-related decreases in the areal size of these areas [29]. Chang *et al.* [29] also found that area V3 was strongly correlated with perceptual learning. It is possible that degradation in area V3 is associated with older adults’ ability to interpret the positional information, which then impacts their ability to attend and inhibit in other conditions. Yet, old-olds were uniquely affected by the presence of the prime and thus an alternative theory is that working memory is also involved in the multistable perception of the old-olds. Research on younger adults has shown that increasing working memory load can decrease the frequency of reversals and increase the response time between reversals (response duration) and working memory load can influence reversal related ERP brain responses to the Necker cube [11,30]. Intaite *et al.* [11] suggests that the decreased amplitudes in the reversal negative (RN) response and the late post component (LPC) responses at P3 are indicative of a larger mental capacity investment. Further, the effect of working memory appeared to affect reversal rates only for continuously presented stimuli. Our oldest-old participants, similar to those in Intaite *et al.*’s [11] high load conditions, show fewer reversals and this may be due to low working memory capacity investment. Allen *et al.* [31,32] found in young adults that working memory (based on reading span) was positively associated with time in the dominant perspective (increases in WM capacity lead to increases in dominant perspective) and negatively associated with reversals during passive viewing (those with the strongest capacity had the lowest reversal rates). Allen *et al.* [32] suggested that working memory is an indicator of the ability to exert attentional control over one’s perspective. In contrast, our participants did not show age related differences in the duration of the down to the left perspective during passive viewing and our oldest-old showed fewer reversals. Here we measured the time in one perspective rather than the average time between reversals (dominant perspective) so that we could make direct baseline comparisons across our four experiments. Time in the dominant perspective does not identify the specific orientation of the cube. It is possible that time in one verses the other orientation is quite different as evidenced by our significantly different reversal rates between older olds and young participants, but similar time in overall down to the left perspective in Experiment1. Our outcome, that older adults show fewer reversals than do younger adults supports previous literature [21,24,25]. Thus, it is surprising that those demonstrated in Allen *et al.*’s [32] study with the strongest WM had the lowest reversal rates. However, this may be due to the difference in sample. All participants in the study conducted by Allen *et al.* [32] were university students, and while some may have lower working memory than others, their working memory, even in the low working memory group, is likely stronger than most of those in our oldest-old group. It is possible, that a difference in Necker response would occur with young adults who show large working memory losses that are comparable to the oldest-old adults. Regardless, our results, which show that the oldest-olds differ from younger adults in several ways leads to the same speculation, that working memory is involved in the processing of multistable stimuli like the Necker Cube.

Working memory plays an important role in inhibitory functions; access, deletion and restrain [2]. GABAergic neurons improve functioning in area V1 and V2, and age-related decreases in the l-glutamic acid decarboxylase (GAD) (*i.e.*, enzymes that synthesize GABA) are thought to be responsible for decreased inhibitory function in the visual system [18,33]. In the case of multistable perception, the visual system must continuously reorganize and interpret the instability of the sensory input, which requires higher order regions. In order to examine the influence of higher order regions in this process, participants in this study were exposed to priming and volition experimental conditions. Only old-olds interpreted the orientation most often in the direction of the prime, which suggests that they did not inhibit the implicit presentation of the prime. On the other hand, younger adults and young-olds showed adaptation effects to the prime cube even with these short exposures. According to Kanai and Verstraten [34], brief exposure to unambiguous stimuli (*i.e.*, 80 ms) leads to visual motion priming (priming effects), while longer exposure (*i.e.*, 320 ms) to unambiguous stimuli leads to an adaptation effect. In multistable perception, perception constantly fluctuates; however, perceptual sensitization (stability in orientation during multistable perception) develops gradually over the course of a few seconds with 3–5 s of blank intervals [34]. In the current study, participants were exposed to an unambiguous prime cube for 5 s (5000 ms) prior to viewing an unambiguous stimulus, and a priming effect was observed only in the old-old group. Based on Kanai and Verstraten’s work [34], a 5000 ms exposure should lead to adaptation, yet, it acted as a prime in the old-old group but not in the young-old group. This suggests that the degree of age-related decline in early visual processing areas and in physiological processes matter and that the old-old group may have reached a critical loss that affects visual inhibition and impacts working memory.

Young-olds and younger adults showed very similar response patterns in all conditions. This supports the idea that changes in multistable perception occur gradually and becomes evident at old-old ages (80+). Such changes may be related to the individuals’ ability to resolve ambiguities in the environment and our data suggest that young-olds are able to do so. However, unlike their counterparts (younger adults or old-old adults) many of the young-old responses were correlated with their visual acuity and stereo-acuity. Overall, young-olds showed adaptation effects when exposed to the prime and this may have been expected as noted above. However, those with the poorest acuity showed the strongest adaptation and this seems counterintuitive. It is important to note that the acuity deficits in the participants in the young-old group were, as expected, minimal with all but one participant having acuities better than 20/40 and one participant with an acuity score of 20/50. In these acuity ranges it is possible to easily resolve our stimuli, however, participants with poorer acuity may increase attention to compensate for minor losses. Such increases in attention to the prime in this case may have increased adaptation effects. In the old-old group acuities were not related to their performance, even though they had a similar range of acuities. Given the presence of other deficits in this age group as described above, they may have different strategies in dealing with ambiguous stimuli.

Those young-old adults with the poorest depth performance showed less ability to inhibit during volition conditions and more intrusions of the opposite orientation after adaptation. Researchers have previously suggested that motion and positional information (such as those in the Necker stimulus) may share common mechanisms [35,36], and that motion and stereopsis share common mechanisms [36,37]. Losses in stereoacuity begin to show after 50 years of age [38] and here we show that these losses impact the ability to inhibit intrusions in our young-old age group only. We believe that this study offers more evidence for the mechanism link between stereovision and motion. Old-old adults also showed stereo losses, but they were only linked to their stability of perception during priming. The difference between the age groups suggests different processing. While we suggest that multistable perception in the old-old age group is impacted by neural noise and deficits in the early visual processing chain, which in turn affects later processing, this is not true for the young-olds. The impact of the stereo deficits on the old-old age group may be masked by other deficits (as described above).

One difficulty in testing older adult populations is their uncontrollable heterogeneity in vision. Our older adults vary on depth, acuity, and contrast ability, among other things. While we tried to control and evaluate many of these differences, it is not possible to control for all potential visual issues in a systematic study and this is a limitation in vision studies with older adults. Regardless, we believe that our study does reveal that our old-old adults uniquely process multistable stimuli and this could not be fully accounted for by the vision variables we tracked (acuity, contrast, depth).

In summary, our experiment shows that old-old adults, unlike their younger counterparts, are influenced in visual construction by external information sources and less able to inhibit external sources during multistable viewing. We suggest this is due to a noisy visual system in combination with neural deficits apparent in the early visual processing which impact higher order processing.

## 5. Conclusions

Our findings suggest that the ability to construct a coherent whole from fragments is exacerbated with age. More specifically, vision is constructed differently in the old-old adults, which can influence environmental interpretations and navigational abilities in this age group. For example, old-old adults’ inability to rapidly solve the ambiguities in their environment could affect their ability to change perspectives or to recognize change quickly when it occurs in the environment (*i.e.*, efficiently processing rapidly changing environments while driving). However, it is important to uncover the neural underpinnings of the behavioral differences examined in this study between age groups in order to characterize and strengthen the causal relationship between multistable perception and age-related changes observed at different levels of processing. Therefore, future research can utilize neuroimaging techniques to strengthen and support the behavioral findings outlined in the present study.

## Figures and Tables

**Table 1:** Mean age and visual demographics and standard error (SE) for younger, young-old and old-old adults.

Group	Age (SE) Years	Acuity (SE) Snellen Equivalent	Letter Contrast (SE) Snellen Equivalent	Depth Disparity (SE) Arch Minutes
Younger	20.12 (0.49)	20/12.84 (0.54)	20/19.08 (1.15)	40 (0)
Young-old	71.4 (0.83)	20/20.84 (1.65)	20/40.48 (7.22)	170.4 (50.2)
Old-old	84.08 (0.66)	20/23.80 (3.34)	20/45.84 (7.31)	195.6 (43.98)
